# Wrack Burial Limits Germination and Establishment of Yellow Flag Iris (*Iris pseudacorus* L.)

**DOI:** 10.3390/plants12071510

**Published:** 2023-03-30

**Authors:** Jesús M. Castillo, Blanca Gallego-Tévar, Brenda J. Grewell

**Affiliations:** 1 Departamento de Biología Vegetal y Ecología, Universidad de Sevilla, Ap. 1095, 41080 Sevilla, Spain; 2 USDA-ARS, Invasive Species & Pollinator Health Research Unit, Department of Plant Sciences MS-4, University of California, 1 Shields Avenue, Davis, CA 95616, USA

**Keywords:** alien invasive species, disturbance ecology, plant invasions, seed quiescence, seedling emergence, tidal marsh, vegetation dynamics, wetland management

## Abstract

Seed burial under wrack, mats of water-transported plant debris, can limit recruitment of seedlings in wetlands. In a greenhouse experiment, we studied the effects of wrack burial (0, 1, 2, 4, 8 cm depths) on germination and emergence of the macrophyte *Iris pseudacorus,* native to Europe, Mediterranean Basin, and western Asia, that has invaded wetlands in nearly every global ecozone. We recorded the percentages of germinating, senescent, and quiescent seeds and evaluated seedling establishment and growth relative to substrate environmental variables. Seedling emergence of *I. pseudacorus* was reduced from >80% in controls without burial to <40% even at minimal wrack depths of 1 cm. Few *I. pseudacorus* seedlings were able to emerge from wrack burial of up to 8 cm in depth. We also found greater numbers of both quiescent seeds and germinated seeds that did not emerge from wrack burial. Reduced seedling emergence and increased seed quiescence with wrack burial were primarily explained by a reduction in daily temperature variation within the substrate. No senescent seedlings were observed with any depth of wrack burial. In view of our results, the management of *I. pseudacorus* invasion will be a long-term challenge, requiring continued control due to persistent seeds in wrack-buried seed banks.

## 1. Introduction

Germination and establishment are crucial stages in plant life cycles and determine community dynamics [1,2]. For example, germination and establishment are key processes during invasions by alien plant species [3,4]. Management of plant invasions can be especially difficult for species that maintain persistent seed banks [5,6]. In this context, burial of seeds at shallow depths can promote the germination, establishment, and formation of seed banks [7]. In fact, some seeds show appendages (awns) that exploit changes in relative humidity for dispersal and self-burial [8]. However, seed burial in sediment in the soil under wrack, stranded mats of water-transported plant debris, may limit the germination and recruitment of seedlings. Thus, some buried seeds germinate only when they are returned to or are near the open soil surface [9,10]. Burial effects on germination may be related to multiple environmental factors, such as poor aeration [11], low temperature fluctuation [12], and/or darkness [13]. Additionally, litter accumulation and decomposition can drive secondary seed dispersal and viability as well as seedling establishment and growth [14,15]. Seed burial under plant litter is especially abundant in ecosystems such as crop lands, peats, coastal forests, and marshes because they can accumulate loads of plant debris [16,17,18].

In tidal marshes, macrophytes show high primary productivity rates that together with low rates of herbivory and decomposition render high quantities of senescent plant material [19]. Floating wrack mats formed primarily from this senescent debris are transported into marshes and redistributed by tides and storm events [20]. Wrack mats are also significant vectors of live seed dispersal into tidal wetlands, influencing plant invasion and vegetation colonization [21] and are a viable source for colonization of developing tidal wetlands as tidal hydrology is restored [22,23]. Plant litter accumulates ashore as mats of wrack that are deposited primarily along drift lines of the highest tides in high-elevation marsh zones where they are trapped by tall vegetation [24,25,26]. Lower tides can also deposit significant amounts of wrack where it can accumulate and persist at lower elevation strandlines, smother vegetation, and eventually decompose and locally expose bare, open habitats [20]. Through this process, tidal deposition of wrack mats is a part of a natural disturbance regime that can temporally limit new vegetation recruitment [27,28,29,30]. When conditions are not favorable, ungerminated seeds remain quiescent and dormant in seed banks [31]. In contrast, the deposition of thin layers of wrack may also promote the colonization of certain macrophytes by ameliorating environmental stress, such as salinity [24], and creating light gaps that promote germination and seed bank emergence [32]. Decomposition of wrack can change the degree of burial over time and alter potential recruitment environments. 

The focus of our study was to analyze the effects of wrack burial on germination and establishment of *Iris pseudacorus* L. (yellow flag iris; Iridaceae). *Iris pseudacorus* is a perennial macrophyte native to Europe, Mediterranean Basin, and western Asia [33] that is invading wetlands in nearly every global ecozone [34,35,36]. *Iris pseudacorus* produces large quantities of seeds that are highly viable, and its invasive spread is almost entirely by hydrochorous dispersal of these buoyant seeds [37]. Fruit capsules of mature *I. pseudacorus* plants often extend from stems arching directly over the water, with seeds dispersing directly into currents after which they can deposit in zones along with wrack mats. 

The mass of *I. pseudacorus* seeds is relatively high, ca. 60–70 mg dry weight (DW), and their maximum germination percentages are achieved in freshwater and light conditions under daily alternating temperatures [38,39,40]. Large-seeded species may be less affected by wrack burial because large-buried seeds that germinate have more reserves needed by seedlings to reach the soil surface [4,41,42]. However, growing through the substrate may be difficult for large seedlings coming from large seeds [7], so large seeds buried deeply may or may not result in high seedling emergence [43,44]. *Iris pseudacorus* seedlings have thin and sharp-edged leaves and leaf tips, which facilitate emergence from soil burial, as reported for the invasive cordgrass *Spartina densiflora* Brongn. [7].

Our aim was to examine the effects of five wrack burial depths (0, 1, 2, 4, and 8 cm) on the germination and establishment of *I. pseudacorus*. To achieve this objective, we evaluated percentages of germinating, quiescent, dormant, and dead seeds and seedling survival and emergence differences of *I. pseudacorus* at various levels of wrack burial under controlled conditions in a greenhouse experiment. Significant environmental factors (substrate pH, electrical conductivity, redox potential, and temperature) that potentially influence germination and establishment of macrophytes in marshes were also measured in experimental treatments. We hypothesized that wrack burial would reduce germination and establishment of *I. pseudacorus* due to low temperature fluctuations and dark conditions resulting from burial by wrack.

## 2. Results

### 2.1. Environmental Conditions

**Figure 1:**
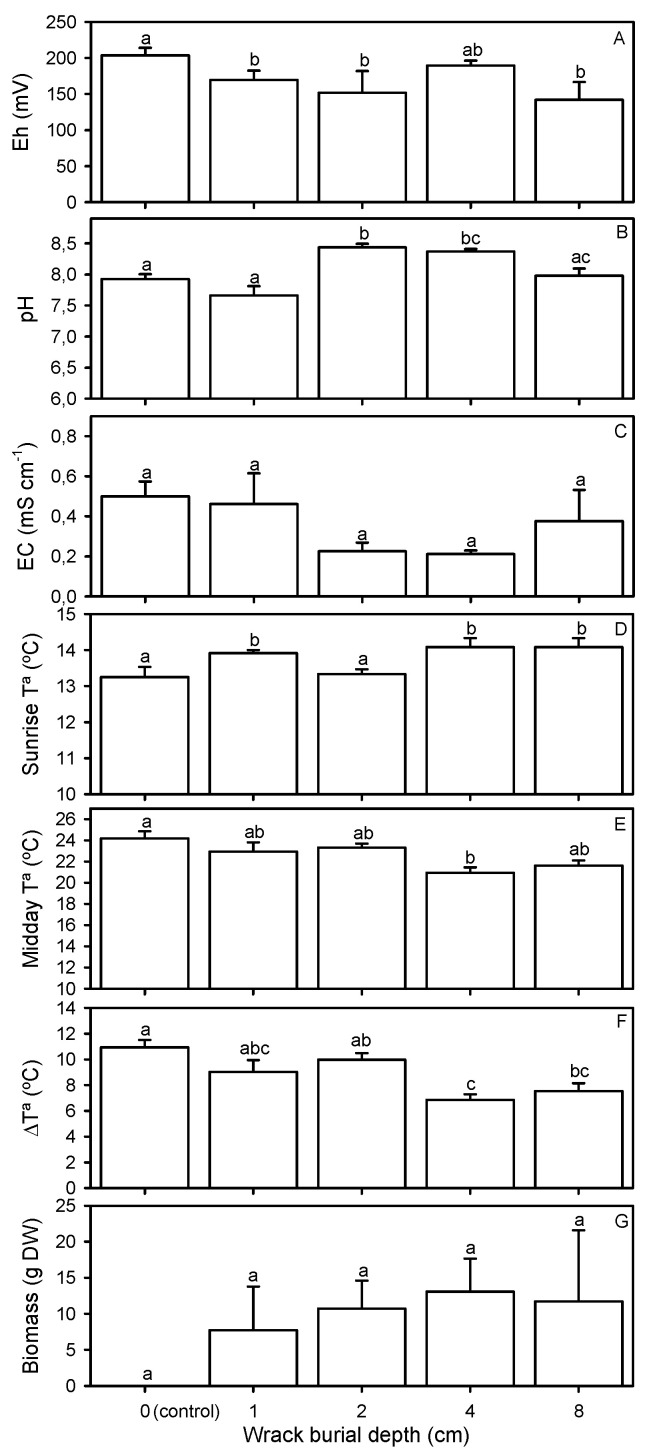
Substrate (**A**) redox potential (Eh), (**B**) pH, (**C**) electrical conductivity (EC), temperature at (**D**) sunrise and (**E**) midday, and (**F**) the difference (Δ) between midday and sunrise temperatures, and (**G**) biomass (in dry weight, DW) of plant species other than *Iris pseudacorus* in the five wrack burial treatments. Values are mean ± SE. Different small letters over the columns indicate significant differences among treatments (LM or GLM, *p* < 0.05).

Mean Eh of the substrate values was always positive, varying between 142 ± 25 mV at 8 cm deep and 204 ± 11 mV at the control treatment (Figure 1A and Table 1). Mean pH of the substrate was between 7.7 ± 0.2 at 1 cm deep and 8.4 ± 0.1 at 2 and 4 cm deep (Figure 1B and Table 1). Substrate pH decreased with higher EC values (Table S1). Substrate EC did not change between burial treatments, being always close to 0.4 mS cm^−1^ (Figure 1C and Table 1). Mean temperature of the substrate at sunrise increased at higher wrack depths (Table S1), varying between 13.3 °C for the control treatment and at 2 cm deep and 14.1 ± 0.3 °C at 4 and 8 cm deep (Figure 1D and Table 1). In contrast, mean temperature at midday decreased at higher wrack depths (Table S1), being between 24.2 ± 0.7 °C for the control treatment and 20.9 ± 0.5 °C at 4 cm deep (Figure 1E and Table 1). Daily variation in temperature also decreased at higher wrack depths (Table S1), being maximal for the control treatment (10.9 ± 0.6 °C) and minimal at 4 cm deep (6.9 ± 0.4 °C) (Figure 1F and Table 1). Air minimum daily temperature was 8.5 °C in February at the beginning of the experiment. Air maximum daily temperature increased during the experiment from 31 °C in February to 41 °C in June at the end of the experiment.

We recorded 13 plant species established from seeds included in the wrack: 11 native species (*Atriplex chenopodioides* Batt., *Bolboschoenus maritimus* (L.) Palla, *Cynodon dactylon* (L.) Pers., *Lotus angustissimus* L., *Oenanthe lachenalii* C.C. Gmel., *Plantago latifolia* L., Poaceae sp., *Polygonum equisetiforme* Sm., *Rumex conglomeratus* Murray, *Taraxacum officinale* (L.) Weber ex F.H.Wigg., *Trifolium repens* L., and *Verbena officinalis* L.) and 2 alien species (*Datura stramonium* L. and *Eclipta prostrata* (L.) L.). Total plant biomass of emergent species other than *I. pseudacorus* was close to 10 g DW for every burial wrack treatment (Figure 1G and Table 1).

### 2.2. Plant Traits

**Figure 2:**
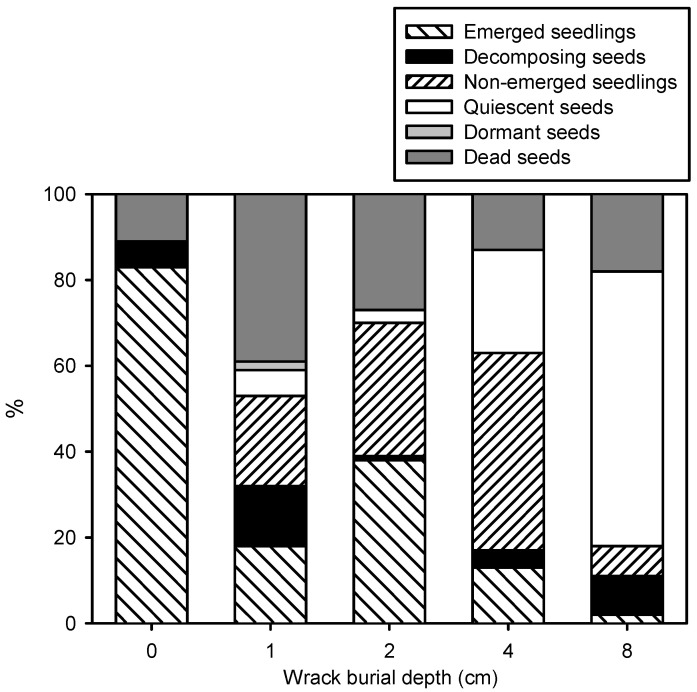
Percentages of seedling emergence, decomposing seeds, non-emerged seedlings, quiescent seeds, dormant seeds, and dead seeds of *Iris pseudacorus* at different wrack burial depths. Values are means.

**Figure 3:**
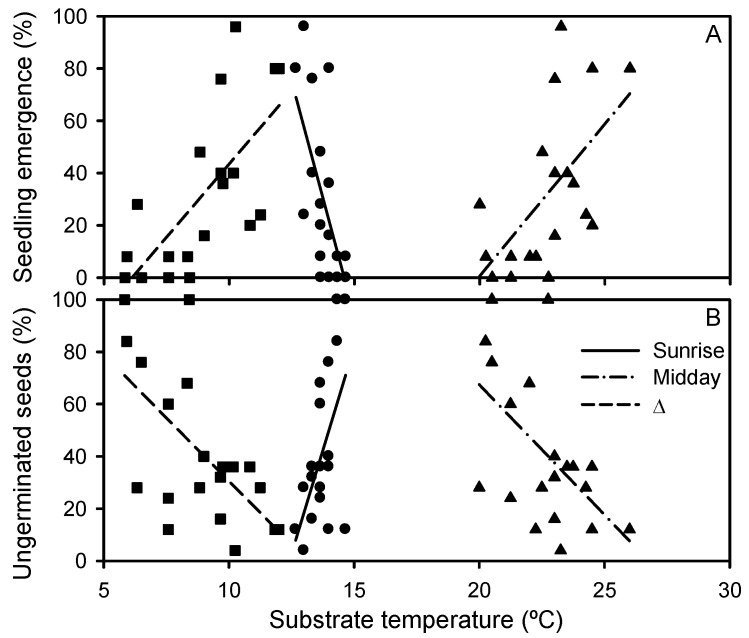
Relationships between the percentages of (**A**) seedling emergence and (**B**) non-decomposing ungerminated seeds of *Iris pseudacorus* with substrate temperatures at sunrise (∂) and midday (▲) and the difference (Δ) between midday and sunrise temperatures (■). Regression equations: (**A**) Sunrise Tª, y = 522.520 − 35.805x, R^2^ = 0.392, *p* = 0.003, and n = 20; Midday Tª, y = −232.141 + 11.635x, R^2^ = 0.377, *p* = 0.004, and n = 20; ΔTª, y = −68.519 + 11.201x, R^2^ = 0.485, *p* = 0.001, and n = 20; (**B**) Sunrise Tª, y = −391.837 + 31.561x, R^2^ = 0.328, *p* = 0.010, and n = 20; Midday Tª, y = 266.783 − 9.964x, R^2^ = 0.298, *p* = 0.013, and n = 20; ΔTª, y = 127.286 − 9.664x, R^2^ = 0.390, *p* = 0.003, and n = 20.

**Figure 4:**
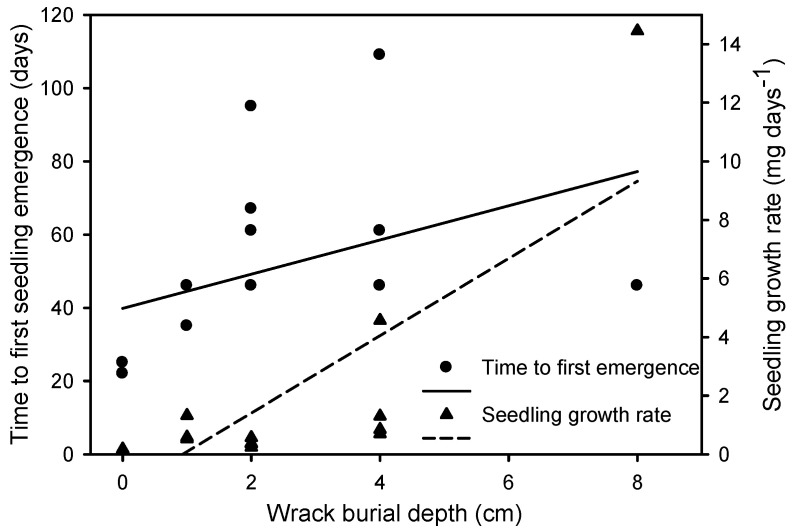
Relationships between the time to first seedling emergence (∂) and seedling growth rate (▲) of *Iris pseudacorus* with wrack burial depth. Regression equations: Time to first seedling emergence, y = 39.851 + 4.668x, R^2^ = 0.170, *p* = 0.004, and n = 16; Seedling growth rate, y = 1.227 + 1.318x, R^2^ = 0.638, *p* < 0.0001, and n = 16.

The percentage of emerged seedlings decreased with increasing wrack depth (β = −0.418), with the highest emergence in the control treatment (83 ± 4%) and lowest under 8 cm of wrack (2 ± 2%) (Figure 2 and Table 1). In addition, the percentage of emerged seedlings decreased at a higher substrate temperature at sunrise (β = −0.697) and increased together with substrate temperature at midday (β = +1.334) and the difference between midday and sunrise temperatures (β = −1.301) (Figure 3A and Table S1). The time to first seedling emergence changed between 24 ± 1 days in control conditions and 99 ± 35 days under 8 cm of wrack (Table 1), increasing together with burial depth (β = 0.432) (Figure 4) and substrate sunrise temperature (β = 2.433). Time to first emergence also decreased with increasing substrate Eh (β = −0.236), midday temperature (β = −6.737), and the difference between midday and sunrise temperatures (β = 7.907) (Table S1). In addition, seedling growth rate increased together with burial depth, varying between 0.16 ± 0.01 mg day^−1^ for the control treatment and 19.22 ± 1.59 mg day^−1^ for the only two plants that emerged as seedlings from a burial of 8 cm deep (Figure 4, Table 1 and Table S1). All emerged and buried seedlings survived to the end of the experiment (Figure S1).

The percentage of decomposing seeds did not change significantly between burial depths, showing mean values between 1 and 14% (Figure 2 and Table 1). The percentage of non-emerged seedlings varied between 0 ± 0% for the control treatment and 46 ± 15% at 4 cm deep (Figure 2 and Table 1), increasing together with the biomass of other plant species (Table S1). The percentage of ungerminated seeds was the lowest in control conditions (11 ± 3%) and the highest at 8 cm deep (82 ± 11%) (Table 1). The percentage of ungerminated seeds increased together with burial depth (β = 0.367) and substrate sunrise temperature (β = −5.414) and decreased with increasing substrate Eh (β = −0.303), midday temperature (β = 16.451), and the difference between midday and sunrise temperatures (β = −19.677) (Figure 3B and Table S1).

### 2.3. Recovery Experiment

No quiescent seeds were observed in experimental control treatments. Only 2 seeds sowed at 2 cm deep and 1 seed at 4 cm deep germinated later than 6 months after sowing. The maximum percentage of quiescence (64 ± 17%) was recorded at 8 cm deep (Figure 2 and Table S1). The percentage of quiescent seeds increased with wrack burial depth (β = 0.617) and substrate temperatures at sunrise (β = −7.269), midday (β = 22.170), and their daily variation (β = −26.272). We recorded only 2 dormant seeds at the end of the recovery experiment coming from burial at 1 cm deep. The percentage of dead seeds did not change significantly between burial depths and was independent of every environmental factor (Figure 2, Table 1 and Table S1).

## 3. Materials and Methods

### 3.1. Seed and Wrack Material

Seeds were collected at the end of the 2018 growing season from randomly selected mature perennial patches of *Iris pseudacorus* colonizing a tidal marsh in the Guadalquivir River Estuary (37°22′36.4″ N, 6°1′16.1″ W) within its native distribution range (Southwest Iberian Peninsula, Andalusia, Spain). Tidal marshes in this estuary experience mixed semidiurnal tidal regime with mesotidal ranges and are under Mediterranean climate with cool, wet winters and hot, dry summers moderated by ocean influence [45]. Plant wrack was collected within one month from the same location where iris seeds were collected. Wrack was composed primarily of plant debris, including senescent *Bolboschoenus maritimus* (L.) Pallas, *Typha* sp., *Arundo donax* L., *Phragmites australis* (Cav.) Trin. ex. Steud., *Cynodon dactylon* (L.) Pers., and *Populus* sp. and *Salix* sp.

### 3.2. Burial Experiment

Our experiment was conducted in the greenhouse facility of the University of Seville at ambient light and air temperature conditions over four months from early February to June 2019 to test the effects of wrack burial on seed germination and seedling emergence. Four replicates of 25 seeds per treatment were sown at 0.5 cm depth in vermiculite in plastic containers (18 cm width, 22 cm length, and 11 cm height) containing 4 cm depth of vermiculite. Five wrack burial treatments were conducted: control (0 cm depth, no wrack was added), 1 cm (2767 ± 65 g DW wrack m^−2^), 2 cm (5975 ± 802 g DW m^−22^), 4 cm (11,921 ± 1365 g DW m^−2^), and 8 cm (21182 ± 1440 g DW m^−2^) of wrack burial depth. In total, 20 containers (5 treatments × 4 replicates) were set up in a randomized complete block design. Because *I. pseudacorus* shows its highest germination percentages under high humidity conditions [39], containers were carefully irrigated twice a week to ensure the substrate remained waterlogged. *Iris pseudacorus* is sensitive to salinity [38]. Therefore, fresh water was used in treatments to avoid salinity effects on germination, because we wanted to assess the responses of seeds and seedlings to burial without potentially confounding effects of salinity. The experiment continued until no additional emergence was observed in the control treatments for at least 30 days. This duration of the experiment was the usual time needed for the germination of *I. pseudacorus* in freshwater conditions [38,39].

### 3.3. Environmental Conditions

Minimum and maximum air temperatures (°C) were recorded using a max/min thermometer (Piao) during the experiment. Substrate temperatures at sunrise and solar midday were recorded using glass alcohol thermometers (MiniScience GAT20110YP), and substrate redox potential (Eh) was recorded using a portable meter and electrode system (Crison pH/mV p-506) monthly in February, March, and April 2019. At the end of the experiment, electrical conductivity (EC; as a record of salinity) of the interstitial water of the substrate was recorded using a conductivity meter (Crison-CM35), and pH was recorded using a pH meter (Crison PH25 with an electrode M-506). Substrate temperatures, Eh, pH, and EC were recorded at 0.5 cm deep in the vermiculite where seeds were sown (n = 12 per treatment for Eh and substrate temperatures and n = 4 per treatment for EC and pH). We also recorded the total biomass of other plant species, in addition to iris, that germinated or re-sprouted from propagules included in the wrack. Biomass was recorded after drying the samples in a forced-air stove at 80 °C for 48 h.

### 3.4. Plant Traits

Seedling emergence through the vermiculate (in the control treatment) and through wrack surface (visible above wrack surface in the burial treatments) was recorded weekly. The time to first seedling emergence was recorded, and it was related to the total duration of the experiment (116 days) when no seedlings had ceased to emerge in any treatment prompting termination of the experiment. At the end of the experiment, the wrack and the vermiculite were carefully removed, and we counted decomposing senescent seeds, germinated seeds (radicle emerged but without emergent seedlings), and ungerminated seeds that were not decomposing. Seed and seedling percentages were calculated in relation to the total number of seeds per replicate. Survivorship was determined for all *I. pseudacorus* seedlings that emerged through the experiment. At the end of the experiment, total live biomass was determined for every surviving emergent *I. pseudacorus* seedling. Seedling growth rate (mg day^−1^) for each replicate was calculated as the quotient between mean biomass and mean growth period after emergence for every iris seedling.

### 3.5. Recovery Experiment

Ungerminated non-decomposing seeds were transferred to fresh water in transparent plastic containers (7.0 cm height and 5.5 cm diameter) for 84 weeks from the end of the experiment (3 June 2019–10 January 2021). This long duration of the recovery experiment was designed to allow all quiescent seeds to germinate when exposed to changing temperatures throughout the seasons. Seeds that germinated during the recovery experiment were considered quiescent as they did not germinate during the burial experiment due to the absence of some environmental factor necessary for germination [46]. The embryo viability of all ungerminated seeds was tested with tetrazolium (2,3,5-triphenyl-2H-tetrazolium chloride) solution at 0.1% at the end of the recovery experiment. Seeds were cut in half to bisect the embryo and submerged in the tetrazolium solution for 24 h at 25 °C. Seeds with viable embryos presented pink or red color, and they were considered dormant. Seeds without dyed embryos were considered dead. Percentages of quiescent, dormant, and dead seeds were calculated in relation to the total number of seeds per replicate.

### 3.6. Statistical Analyses

Analyses were carried out using SPSS release v. 12.0 for Windows (SPSS Inc., Chicago, IL, USA), applying a significance level (α) of 0.05. Deviations were calculated as standard errors (SEs). Data series were tested for normality and homogeneity of variance using Kolmogorov–Smirnov and Levene tests, respectively. Biomass of plant species was transformed using the function √x to achieve homogeneity of variance. We ran analyses of correlation (Pearson correlation coefficient, r) and regression (coefficient of determination, R^2^) to investigate the relationships between environmental factors and plant traits. When a plant trait was correlated with two or more environmental factors, multiple regression analysis was carried out to explore relative weights (β). We ran a General Linear Model (LM; F-test) to analyze differences in environmental factors and plant traits (dependent variables) between burial treatments (fixed effect) with Tukey’s Honest Significant Difference (HSD) test as post hoc test. Substrate Eh, seedling growth rate, and dormant seed percentage were transformed using the functions 1/x, √x, and ln (x) trying to achieve normality or homogeneity of variance. Because normality or homoscedasticity were not achieved after data transformation, univariate differences between burial treatments were analyzed using the Gamma Generalized Linear Model (GLM) with Chi-square (χ^2^) of Wald.

## 4. Discussion

Our results show that wrack burial limits germination and seedling emergence of *I. pseudacorus*. Seed burial at shallow depths can stimulate germination and establishment by maintaining a moist environment around seeds and roots, preventing desiccation [41,47]. However, seedling emergence of *I. pseudacorus* was reduced from more than 80% without wrack burial to less than 40% at wrack depths of even just 1 cm, which was related mainly to a reduction in daily temperature variation within the substrate. *Iris pseudacorus* shows increases in its germination percentage by ca. 70% from daily constant to alternating temperatures [39]. The drastic limitation in seedling emergence we observed occurred even though *I. pseudacorus* seedlings emerged along a single axis, relying on the elongation of the mesocotyl, an anatomical feature of monocots. In *Spartina alterniflora* Loisel., a macrophyte colonizing intertidal marshes as *I. pseudacorus*, the mesocotyl has been shown to raise the coleoptilar node above the soil surface, which has been interpreted as an important adaptation in anoxic salt marsh soils [48].

The reduction in seedling emergence in wrack burial treatments was accompanied by increases in both quiescent seeds and seedlings that did not emerge through wrack though their seeds had germinated under wrack. The quiescent seed percentage was maximum (64%) at 8 cm deep burial under wrack, whereas non-emerged seedlings were maximum (46%) where burial was 4 cm deep. No dead seedlings were recorded at any wrack burial depth, which was probably related to sufficient carbohydrate reserves in *I. pseudacorus* seeds [49]. These seed reserves would support seedling growth through the hollows left open in the wrack (reflected in curved mesocotyl) and survive while buried with the potential to emerge as bare soil is exposed when wrack decomposes or is removed. The ability of a few *I. pseudacorus* seedlings to emergence from wrack burial depths as high as 8 cm contrasts with other emergent monocot species, such as *Spartina densiflora* Brongn., a wetland grass that has much smaller seeds and is not able to emerge from wrack depths higher than 1 cm [50]. Moreover, wrack burial increased the time to first seedling emergence of *I. pseudacorus*, which could be related to a longer time lapse to grow through the wrack from deeper depths and to an increase in the germination period of this species at lower temperatures [39]. Additionally, the seedling growth rate increased with wrack burial depth as reported previously for sediment burial due to stimulation of root growth that could help seedlings absorb more nutrients [51]. In this sense, moderate wrack burial would reduce seedling establishment percentage but, at the same time, it may stimulate the invasion of exotic *I. pseudacorus* by promoting the growth of the few plants able to survive.

The increase in seed quiescency we recorded at higher wrack burial depths was not related to poor aeration conditions [52] because substrate Eh was higher than +140 mV at every wrack depth in our experiment. In fact, the recorded increase in seed quiescency with wrack burial was mainly related to a lower variation in daily substrate temperature. In this sense, germinating at fluctuating temperatures may act as a mechanism by which seeds detect gaps in vegetation canopies and avoid germinating too deep in the soil [44,53]. In our study, the daily temperature variation in the substrate below the wrack depended markedly on the substrate temperature at midday that increased to values higher than 25 °C and without wrack, close to the optimum 28 °C germination temperature for *I. pseudacorus* [39]. Accordingly, *I. pseudacorus* shows its maximum germination percentages at alternating temperatures [39]. In addition, [39] also described that *I. pseudacorus* is able to germinate at temperatures as high as 36 °C, which would explain the germination of quiescent seeds in the recovery experiment, once the limitations imposed by wrack burial were removed. In addition to daily temperature variations in the substrate, wrack burial may have also diminished germination by reducing the light intensity [54], because darkness conditions reduced *I. pseudacorus* germination by ca. 15% [39]. Furthermore, allelopathic effects from the plant debris inhibiting the germination of *I. pseudacorus* cannot be ruled out. In any case, the ability of *I. pseudacorus* to bank its quiescent seeds is consistent with observations by [7,55] of the tendency for effective colonizers rather than later successional species to bank seeds in tidal marshes.

Our results are useful to understand the colonization process of *I. pseudacorus*. For example, the establishment of *I. pseudacorus* would be temporally limited by wrack burial in intertidal zones where wrack is primarily deposited along tide strand lines [56]. In these environmental conditions, wrack accumulation could provide a degree of seed quiescency and at greater wrack depths would limit the establishment of *I. pseudacorus*, and the wrack resulted in up to 14% of seeds undergoing decomposition below, which may be caused by fungal infection and/or an unfavorable microenvironment [10]. However, many quiescent seeds may survive in the seed banks under mats and play a future role in vegetation dynamics [57]. The ability of alien species to bank seeds can contribute to invasion success, because seeds can persist in unfavorable conditions, especially in environments where opportunities for seed germination are unpredictable, such as tidal marshes [58].

It is important to consider management implications surrounding wrack influence on *I. pseudacorus* colonization dynamics, given its impact on the severe reduction in native macrophyte species diversity in tidal marshes where it is a successful alien invasive species [59]. Our results show that wrack burial limits germination and emergence of *Iris pseudacorus,* yet quiescent seeds persist in the seed banks despite wrack burial. Decomposition or removal of wrack can open habitats and stimulate emergence from the seed banks. In view of our results, the management of *I. pseudacorus* invasion will be a long-term challenge where wrack is present, requiring continued control due to disturbance cycles of wrack deposition and decomposition that bury and subsequently create open habitats that can provide open windows for the invasive plant to emerge from wrack-buried seed banks. In sensitive zones impacted by wrack, strategic removal of wrack mats should be considered in an integrated management scheme to accelerate the depletion of invasive plant seed banks and remove emergent seedlings at a life stage when control is most feasible.

## Figures and Tables

**Table 1:** Results of linear model (LM) and generalized linear model (GLM) for environmental variables and plant traits of *Iris pseudacorus* with wrack burial depth as fixed factor. d.f., degree of freedom.

	LM or GLM (d.f. = 4)
Environmental variables	
Substrate redox potential (mV)	χ^2^ = 10387.300, *p* < 0.0001
Substrate pH	F = 11.427, *p* < 0.0001
Substrate electrical conductivity (mS cm^−1^)	F = 1.323, *p* = 0.306
Substrate temperature at sunrise (°C)	F = 3.943, *p* = 0.022
Substrate temperature at midday (°C)	F = 4.473, *p* = 0.014
Daily temperature variation in the substrate (°C)	F = 7.236, *p* = 0.002
Biomass of plant species (g) (transformed by √x)	F = 2.114, *p* = 0.130
Plant traits	
Seedling emergence (%)	F = 39.703, *p* < 0.0001
Time to first seedling emergence (days)	F = 3.558, *p* = 0.031
Seedling growth rate (mg day^−1^)	χ^2^ = 277.010, *p* < 0.0001
Decomposing seeds (%)	F = 1.137, *p* = 0.377
Germinated and non-emerged seeds (%)	F = 5.522, *p* = 0.006
Non-decomposing and ungerminated seeds (%)	F = 7.233, *p* = 0.002
Quiescent seeds (%)	χ^2^ = 39.187, *p* < 0.0001
Dormant seeds (%)	χ^2^ = 16.000, *p* < 0.003
Dead seeds (%)	F = 2.839, *p* = 0.062

## Data Availability

The data that support the findings of this study are available from the corresponding author, J.M.C., upon reasonable request.

## Supplementary Materials

The following supporting information can be downloaded at: https://www.mdpi.com/article/10.3390/plants12071510/s1, Table S1: Bilateral correlations (Pearson correlation coefficient (r), P, and n) between environmental conditions and plant traits of *Iris pseudacorus* germination and establishment at different wrack burial depths in experimental treatments. Figure S1. (A) *Iris pseudacorus* seedling emerging through the wrack in an experimental treatment and (B) established seedlings of *Iris pseudacorus* and other plant species that germinated or re-sprouted from propagules included in the wrack experimental treatment.
