# Supplementary material for: Wrack Burial Limits Germination and Establishment of Yellow Flag Iris (Iris pseudacorus L.)
Source: Plants (Basel). 2023 Mar 30;12(7):1510. doi: 10.3390/plants12071510 (PMC10096986; doi:10.3390/plants12071510)
Supplement: Supplementary file 1 [file plants-12-01510-s001.zip › Figure S1.pdf]

Supplemental materials

Wrack Burial Limits Germination and Establishment of Yellow Flag Iris (*Iris pseudacorus* L.)

Jesús M. Castillo, Blanca Gallego-Tévar, Brenda J. Grewell

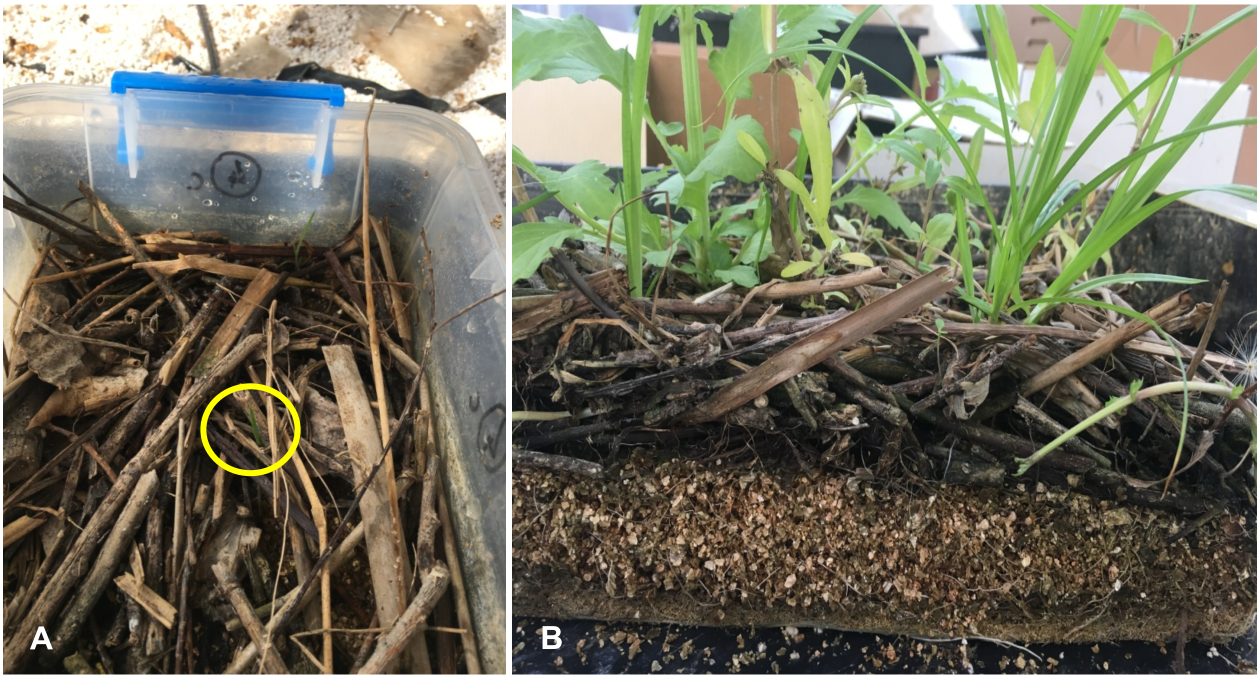

Figure S1. (A) *Iris pseudacorus* seedling emerging through the wrack in an experimental treatment, and (B) established seedlings of *Iris pseudacorus* and other plant species that germinated or re-sprouted from propagules included in the wrack experimental treatment.
