# Supplementary material for: Wrack Burial Limits Germination and Establishment of Yellow Flag Iris (Iris pseudacorus L.)
Source: Plants (Basel). 2023 Mar 30;12(7):1510. doi: 10.3390/plants12071510 (PMC10096986; doi:10.3390/plants12071510)
Supplement: Supplementary file 1 [file plants-12-01510-s001.zip › Table S1.pdf]

Supplemental materials

Wrack Burial Limits Germination and Establishment of Yellow Flag Iris (*Iris pseudacorus* L.)

Jesús M. Castillo, Blanca Gallego-Tévar, Brenda J. Grewell

Table S1. Bilateral correlations (Pearson correlation coefficient (r), P, n) between environmental conditions and plant traits of *Iris pseudacorus* germination and establishment at different wrack burial depths in experimental treatments. Significant correlations are marked in bold.

	Substrate Eh	Substrate pH	Substrate EC	Substrate sunrise temperature	Substrate midday temperature	Daily substrate temperature variation	Biomass of plant species	Emerged seedlings (%)	Days to first emergence	Seedling growth rate	Decomposing seeds (%)	Non-emerged seedlings (%)	Ungerminated seeds (%)	Quiescent seeds (%)	Dormant seeds (%)	Dead seeds (%)
Wrack burial depth	-0.351	.0149	-0.195	**0.495**	**-0.566**	**-0.621**	0.289	**-0.709**	**0.619**	**0.799**	0.015	0.026	**0.710**	**0.795**	-0.236	-0.160
	0.129	0.530	0.410	**0.026**	**0.009**	**0.003**	0.217	**0.000**	**0.004**	**0.000**	0.949	0.912	**0.000**	**0.000**	0.317	0.499
	20	20	20	**20**	**20**	**20**	20	**20**	**20**	**16**	20	20	**20**	**20**	20	20
Substrate Eh	1	-0.100	0.358	-0.356	0.316	0.370	0.066	0.362	**-0.459**	-0.051	0.118	0.211	**-0.584**	-0.442	-0.040	-0.242
		0.675	0.121	0.123	0.175	0.109	0.783	0.117	**0.042**	0.851	0.620	0.371	**0.007**	0.051	0.865	0.303
		20	20	20	20	20	20	20	**20**	16	20	20	**20**	20	20	20
Substrate pH		1	**-0.662**	0.000	-0.095	-0.082	-0.024	0.019	0.047	-0.057	**-0.534**	0.393	-0.129	0.026	**-0.607**	-0.238
			**0.001**	1.000	0.690	0.731	0.918	0.938	0.844	0.833	**0.015**	0.087	0.586	0.912	**0.005**	0.313
			**20**	20	20	20	20	20	20	16	**20**	20	20	20	**20**	20
Substrate EC			1	-0.268	0.213	0.257	**0.483**	0.198	-0.208	-0.263	0.319	-0.158	-0.195	-0.176	0.408	-0.061
				0.254	0.366	0.273	**0.031**	0.403	0.378	0.325	0.170	0.506	0.410	0.458	0.074	0.799
				20	20	20	**20**	20	20	16	20	20	20	20	20	20
Substrate sunrise temperature				1	-0.427	**-0.646**	0.031	**-0.633**	**0.500**	0.237	-0.102	0.169	**0.563**	**0.542**	0.165	0.011
					0.060	**0.002**	0.898	**0.003**	**0.025**	0.377	0.670	0.476	**0.010**	**0.014**	0.487	0.965
					20	**20**	20	**20**	**20**	16	20	20	**20**	**20**	20	20
Substrate midday temperature					1	**0.966**	-0.304	**0.611**	**-0.458**	-0.232	0.073	-0.152	**-0.546**	**-0.449**	-0.184	-0.149
						**0.000**	0.193	**0.004**	**0.042**	0.387	0.759	0.522	**0.013**	**0.047**	0.437	0.532
						**20**	20	**20**	**20**	16	20	20	**20**	**20**	20	20
Daily substrate						1	-0.261	**0.697**	**-0.529**	-0.267	0.094	-0.171	**-0.627**	**-0.541**	-0.200	-0.126

temperature variation							0.266<br>20	**0.001**<br>**20**	**0.017**<br>**20**	0.318<br>16	0.693<br>20	0.470<br>20	**0.003**<br>**20**	**0.014**<br>**20**	0.399<br>20	0.598<br>20
Biomass of plant species							1	-0.326<br>0.161<br>20	0.246<br>0.297<br>20	-0.166<br>0.538<br>16	0.221<br>0.350<br>20	**0.452**<br>**0.045**<br>**20**	-0.065<br>0.784<br>20	-0.030<br>0.900<br>20	0.124<br>0.602<br>20	-0.073<br>0.761<br>20
Emerged seedlings (%)								1	**-0.706**<br>**0.001**<br>**20**	-0.419<br>0.106<br>16	-0.211<br>0.371<br>20	-0.331<br>0.154<br>20	**-0.721**<br>**0.000**<br>**20**	**-0.540**<br>**0.014**<br>**20**	-0.254<br>0.280<br>20	-0.294<br>0.209<br>20
Days to first emergence									1	0.160<br>0.555<br>16	0.057<br>0.810<br>20	0.178<br>0.453<br>20	**0.583**<br>**0.007**<br>**20**	**0.499**<br>**0.025**<br>**20**	0.121<br>0.613<br>20	0.128<br>0.590<br>20
Seedling growth rate										1	0.067<br>0.806<br>16	0.100<br>0.714<br>16	0.458<br>0.074<br>16	0.327<br>0.216<br>16	-0.085<br>0.756<br>16	0.353<br>0.179<br>16
Decomposing seeds (%)											1	-0.156<br>0.511<br>20	0.010<br>0.966<br>20	-0.086<br>0.719<br>20	0.116<br>0.627<br>20	0.168<br>0.479<br>20
Non-emerged seedlings (%)												1	-0.347<br>0.134<br>20	-0.339<br>0.144<br>20	0.047<br>0.845<br>20	-0.008<br>0.974<br>20
Ungerminated seeds (%)														**0.835**<br>**0.000**<br>**20**	0.190<br>0.423<br>20	0.260<br>0.269<br>20
Quiescent seeds (%)														1	-0.198<br>0.402<br>20	-0.313<br>0.179<br>20
Dormant seeds (%)															1	**0.634**<br>**0.003**<br>**20**
